# Long-term outcome of medullary thyroid carcinoma in patients with normal postoperative medical imaging

**DOI:** 10.1038/sj.bjc.6600930

**Published:** 2003-05-13

**Authors:** G Pellegriti, S Leboulleux, E Baudin, N Bellon, C Scollo, J P Travagli, M Schlumberger

**Affiliations:** 1 Service de Médecine Nucléaire et de Cancérologie Endocrieme, Institut Gustave Roussy, Villejuif, France; 2 Département de Biostatistique et de Santé Publique, Institut Gustave Roussy, Villejuif, France; 3 Service de Chirurgie Générale, Institut Gustave Roussy, Villejuif, France

**Keywords:** medullary thyroid cancer, calcitonin, prognostic factors, relapse-free survival

## Abstract

Imaging-detected relapses are observed in a significant proportion of patients with medullary thyroid carcinoma (MTC) with normal postoperative imaging studies. The aim of this study was to search for prognostic factors of imaging-detected relapse. This retrospective study was performed in 63 consecutive MTC patients with normal postoperative medical imaging. After surgery, the basal calcitonin (CT) level was undetectable in 35 patients and elevated in 28. During follow-up, 18 patients developed a clinical or imaging-detected relapse (29%) in the neck and/or at distant sites: 15 had an elevated postoperative basal CT level and three had an undetectable postoperative basal CT level. At multivariate analysis, the significant parameters predictive of imaging-detected relapse were the postoperative plasma CT level and the tumour extension (pT). The 3- and 5-year relapse-free survival rates were 94 and 90% in patients with an undetectable postoperative basal CT level, and 78 and 61% in patients with a detectable basal CT level (*P*<0.05). The 3- and 5-year relapse-free survival rates were 92 and 85% in the pT1–3 patients, and 57 and 46% in the pT4 patients (*P*<0.01). These results show that postoperative CT level and tumour extension are critical prognostic factors for the identification of patients at a high risk of relapse.

Even following apparently complete surgical resection of medullary thyroid carcinoma (MTC), the postoperative plasma calcitonin (CT) level remains elevated in a large proportion of patients. Elevated plasma CT indicates the persistence of neoplastic foci. Furthermore, rare relapses have been reported in patients with an undetectable basal CT after initial surgery (Modigliani *et al*, 1998).

In patients with elevated CT levels, the sensitivity of imaging methods (including ultrasonography, computed tomography scan, MRI or scintigraphy) for the detection of residual disease is limited for lesions other than gross residual disease (Frank-Raue *et al*, 1992). Selective venous sampling catheterisation with plasma CT measurements appears to be the most sensitive and specific method for localising persistent disease. However, even after further surgery based on the results of venous sampling, 62–98% of the patients still have elevated CT levels (Frank-Raue *et al*, 1992; Abdelmoumene *et al*, 1994; Moley *et al*, 1998; Kebebew *et al*, 2000b).

Five and 10-year overall survival rates in MTC patients without initial distant metastases range between 60–100% and 55–100%, respectively, suggesting that an aggressive therapeutic approach may not be appropriate in all patients (Rougier *et al*, 1983; Saad *et al*, 1984; Schroder *et al*, 1988; Raue *et al*, 1993; Dottorini *et al*, 1996; Scopsi *et al*, 1996; Bergholm *et al*, 1997; Modigliani *et al*, 1998). In patients with elevated plasma CT level and with no evidence of gross residual disease after initial surgery, the rate of clinical or imaging-detected relapses is about 40% during the subsequent follow-up (Rougier *et al*, 1983; Van Heerden *et al*, 1990). However, prognostic factors for relapse have not been clearly identified in these patients. The aims of our single centre retrospective study were (1) to describe the long-term outcome of MTC patients without clinically and imaging-detectable disease after initial neck surgery and (2) to search for prognostic factors for imaging-detected relapse.

## METHODS

### Patients

Imaging investigations (including a neck ultrasonography, a chest computed tomography scan, an abdominal ultrasonography or an abdominal computed tomography scan) were performed after initial treatment in 122 MTC patients at the Institut Gustave Roussy between 1988 and 1998. A bone scintigraphy was also performed in patients with elevated postoperative CT levels. Postoperative imaging was abnormal in 59 patients and normal in 63 patients. These 63 patients constitute the population in this retrospective study. Pathologic diagnoses were all reviewed by a single pathologist (B Caillou, Institut Gustave Roussy). Medullary Thyroid Carcinomas were classified as either sporadic or hereditary according to the familial history and the RET proto-oncogene analysis.

Total thyroidectomy was performed in all patients. A complete lymph node neck dissection (LND) was defined as a dissection of bilateral central and lateral lymph nodes; otherwise it was considered incomplete. Tumours were defined according to the tumour–node–metastasis (TNM) classification (Hermanech and Sobin, 1992, p 35) (T stands for the tumour size: T1: diameter⩽1 cm; T2: diameter>1 cm and ⩽4 cm; T3: diameter>4 cm; T4: any size but extension beyond the thyroid capsule; N stands for the lymph node metastases: N0–without and N1–with lymph node metastases; M stands for distant metastases: M0–without and M1–with distant metastases). According to T, N and M, four stages are individualised: stage I: T1N0M0; stage II: T2–4N0M0; stage III: T1–4N1M0; stage IV: T1–4N0–1M1). In 22 patients, more than one surgical procedure was performed within 18 months for incomplete LND and elevated postoperative CT. Follow-up started after the last surgical procedure. Eleven patients received postoperative external radiation therapy to the neck.

Between 6 weeks and 3 months after surgery, plasma CT (ELSA-CT, CIS-Bio International, Gif-sur-Yvette, France; normal range <10 pg ml^−1^) was measured. In 27 of the 35 patients with an undetectable basal plasma CT, a pentagastrin stimulation test was performed (slow intravenous injection of 0.5 *μ*g kg^−1^ pentagastrin (Peptavlon, Wyeth-Ayerst Lab; Philadelphia, PA, USA) over 3 min with CT measurements before and then 3 and 5 min thereafter). Serum carcinoembryogenic antigen (CEA) (Enzymum-test CEA, Boehringer, Mannheim, Germany; normal <7 ng ml^−1^) was measured more than 2 months after surgery.

Patients were then followed up every 6 months, with a clinical examination and measurement of CT and CEA levels. An imaging work-up, consisting of a neck and abdominal ultrasonography, was performed yearly. In case of elevated CT level, a chest computed tomography scan and a bone scintigraphy were also performed yearly. Imaging-detected relapse was defined as an abnormal clinical or imaging examination.

### Statistics

Follow-up ended on 1 January 2002. Three- and 5-year relapse-free survival rates were calculated using the Kaplan–Meier method (Kaplan and Meier, 1958). The Rothman method (Rothman, 1978) was used to calculate the confidence interval (CI).

Prognostic factors for imaging-detected relapse were studied by using univariate log-rank tests and multivariate Cox model analyses; proportional hazard assumptions were checked using graphical methods. The following parameters were compared: age at diagnosis, gender, hereditary status, stage of the disease, tumour extension (pT) (pT4 *vs.* pT1–3), node status (pN), percentage of metastatic lymph node (>10% *vs* <10% of the total number of resected lymph nodes), postoperative basal plasma CT level (detectable *vs* undetectable), external radiation therapy to the neck and the extent of LND (complete *vs* incomplete). As the stage of the disease and the percentage of metastatic lymph node are closely linked to pT and pN, and are therefore competitive factors, multivariate analysis was performed with only three factors: pT, pN and postoperative basal plasma CT level.

## RESULTS

### Patients' characteristics

Sixty-three consecutive patients were included in the study (35 females, 28 males; Table 1

**Table 1:** Characteristics of the 63 patients with normal postoperative imaging

	**All patients**	**Patients without relapse (*n*=45; 71%)**	**Patients with relapse (*n*=18; 29%)**
Mean age (y)(range)	42 (3–72)	42 (3–7)	42 (11–72)
Gender: male/female	28/35	17/28	11/7
Sporadic/Hereditary MTC	44/19	30/15	14/4
(FMTC/MEN 2A/MEN 2B)	(6/9/4)	(6/8/1)	(0/1/3)
Mean follow-up (years) (range)	6.9 (0.4–13.7)	6.6 (0.4–12.6)	7.7 (1.8–13.7)
			
*TNM stage*			
I	10	10	0
II	13	12	1
III	40	23	17
Postoperative CT level: undetectable/elevated	35/28	32/13	3/15
Lymph node dissection: complete/incomplete/not done	42/19/2	30/13/2	12/6/0
Neck radiation therapy	11	7	4

Abbreviations: MTC=medullary thyroid carcinoma; MEN=multiple endocrine neoplasia; FMTC=familial MTC. TNM=tumour – node – metastasis.

). The mean age at diagnosis was 42 years (range, 3–72 years). Medullary thyroid carcinoma was sporadic in 44 patients and hereditary in 19 patients (familial MTC (FMTC), 6; multiple endocrine neoplasia type 2A (MEN 2A), 9; multiple endocrine neoplasia type 2B (MEN 2B), 4). The mean follow-up was 6.9 years (range, 0.4–13.7 years).

A cervical LND was performed in 61 patients and was complete in 43. Two patients underwent a thyroidectomy without LND for a benign thyroid nodule, and a micro-MTC (4 and 5 mm in diameter, respectively) was discovered at the final pathological examination. Postoperative basal CT levels were undetectable and no further surgery was performed in these two cases.

Stage I MTC (T1N0M0) was diagnosed in 10 patients, stage II (T2N0M0) in 13 patients and stage III in 40 patients (4 T1N1M0; 25 T2N1M0; 1 T3N1M0; 10 T4N1M0).

### Postoperative CT and CEA levels

After surgery, basal plasma CT was elevated in 28 patients (44%) (median: 131 pg ml^−1^; range: 12–3772 pg ml^−1^), whereas it was undetectable in the other 35 patients. A pentagastrin stimulation test was performed in 27 of these 35 patients and yielded a detectable stimulated CT level in five (median: 125 pg ml^−1^; range: 43–808 pg ml^−1^). Carcinoembryogenic antigen, measured in 52 patients, was moderately elevated in six (mean: 20 ng ml^−1^; range: 9–26 ng ml^−1^). All patients with an elevated postoperative CEA level also had an elevated postoperative basal CT level (mean CT: 918 pg ml^−1^; range: 26–2000). The mean follow-up was equivalent in patients with undetectable and elevated postoperative basal CT levels.

### Outcome

The 3- and 5-year relapse-free survival rates were 86% (CI, 75–93%) and 79% (CI, 66–87%), respectively. An imaging-detected relapse occurred in 18 patients (11 males, seven females) (29%) (Table 2

**Table 2:** Characteristics of the 18 patients with relapse

	**Gender (age)**	**Lymph neck node dissection**	**Postoperative CT**	**Postoperative CEA**	**Stage (TNM)**	**Hereditary MTC**	**External radiation therapy**	**Time before relapse**	**Site of relapse**	**CT at the time of relapse**	**CEA at the time of relapse**	**Alive (follow-up years)**
1	F (49)	Complete	<10	1	III (T2N1)	Yes (MEN 2A)	No	1.3	Bone	36	2	No (1.8)
2	F (66)	Incomplete	<10	2	III (T4N1)	No	No	3.6	Cervical	20	nd	Yes (11.5)
3	M (62)	Incomplete	<10	<7	III (T2N1)	No	No	2.7	Cervical	nd	nd	Yes (7.5)
4	M (54)	Complete	16	1	III (T4N1)	No	No	4.5	Liver	6290	7	Yes (7)
5	F (11)	Complete	34	2	III (T2N1)	Yes (MEN 2B)	No	4.9	Cervical	134	<2	Yes (5.8)
6	M (19)	Complete	39	2	III (T4N1)	Yes (MEN 2B)	No	5.0	Lung	125	4	Yes (5)
7	M (40)	Complete	40	3	III (T2N1)	No	No	2.1	Bone	500	10	No (6.9)
8	M (36)	Complete	60	<7	III (T2N1)	No	No	9.0	Cervical	nd	nd	Yes (9.5)
9	M (72)	Incomplete	117	nd	III (T2N0)	No	No	6.1	Cervical	1364	3	No (6.4)
10	M (44)	Complete	129	nd	III (T2N1)	No	Yes	7.3	Liver	598	13	Yes (12.6)
11	M (52)	Incomplete	134	<7	III (T2N1)	No	Yes	5.2	Liver	698	6	Yes (13.2)
12	F (48)	Complete	158	6	III (T3N1)	No	No	3.6	Cervical	741	13	Yes (8.1)
13	M (46)	Incomplete	175	nd	III (T4N1)	No	No	2.5	Cervical	1312	16	Yes (7)
14	F (26)	Complete	228	nd	III (T4N1)	Yes (MEN 2B)	No	1.3	Cervical+liver+bone	2220	175	No (3.7)
15	F (65)	Complete	266	17	III (T2N1)	No	No	1.7	Cervical+liver	121	15	Yes (5)
16	F (25)	Incomplete	300	nd	III (T4N1)	No	Yes	6.0	Liver	1350	300	No (13.7)
17	M (41)	Complete	1500	26	III (T4N1)	No	Yes	2.2	Liver	5090	39	Yes (10)
18	M (26)	Complete	3772	6	III (T2N1)	No	No	0.6	Cervical	3100	9	Yes (4)

Abbreviations: MTC=medullary thyroid carcinoma; MEN=multiple endocrine neoplasia; CT=calcitonin; CEA=carcinoembryonic antigen; nd=not done.

). Imaging-detected relapses were located in the neck lymph nodes in eight patients, at distant sites in eight, and in both the neck and at distant sites in two. An isolated neck imaging-detected relapse developed in previously dissected areas in six patients and in areas that had not been dissected in the other two. Isolated distant metastases were found in the liver (five patients), in the chest (one patient) and in bones (two patients). In the remaining two patients, a neck imaging-detected relapse occurred in previously dissected lymph node areas with liver metastases in one and with liver and bone metastases in the other. Fifteen had an elevated postoperative CT level and three had an undetectable postoperative CT level. At the time of imaging-detected relapse, basal CT level was below 150 pg ml^−1^ in five patients (mean: 1481 pg ml^−1^; range: 20–6290) and CEA level was normal in five patients (mean: 41 ng ml^−1^; range: 0–300).

The 3- and 5-year overall survival rates were 98% (CI, 91–100%) and 97% (CI, 88–99%), respectively. Five patients died. One patient with T2N0M0 disease presented a neck relapse after 6.1 years, underwent further surgery and died of respiratory failure during the postoperative period. The other four patients died from distant metastases after 1.8, 3.7, 6.9 and 13.7 years, respectively. They all had stage III disease (two T2N1M0, two T4N1M0). Three had an elevated postoperative basal CT level and one had an undetectable postoperative basal CT level with a pentagastrin-stimulated CT level at 162 pg ml^−1^.

### Prognostic factors for imaging-detected relapse

Univariate analysis showed that the following parameters were significantly predictive of imaging-detected relapse (Table 3

**Table 3:** Prognostic factors for relapse

	***n***	**3-year relapse-free survival (%)**	**CI 95%**	**5-year relapse-free survival (%)**	**CI 95%**	***P***
*Stage*						
I	10	100	–	100	–	0.003
II	13	100	–	100	–	
III	40	78	[63–89]	61	[44–76]	
						
*Tumour extension*						
pT1–T3	53	92	[81–87]	85	[72–93]	0.0003
pT4	10	57	[28–82]	46	[20–74]	
						
*Node*						
N0	23	100	–	100	[68–87]	0.0006
N1	40	78	[63–89]	65	[44–80]	
						
*Postoperative CT level*						
Undetectable	35	94	[80–98]	90	[75–97]	0.0009
Detectable	28	78	[59–90]	61	[41–77]	
						
*Percentage of lymph node metastases*						
<10%	28	100	–	90	[68–87]	0.004
⩾10%	26	72	[53–86]	64	[44–80]	
						
*Gender*						
Female	35	88	[72–95]	81	[64–91]	0.089
Male	28	85	[67–94]	76	[66–88]	
						
*Age at diagnosis*						
<45 years	31	83	[66–93]	79	[60–90]	0.53
⩾45 years	32	90	[74–97]	79	[61–90]	
						
*Hereditary disease*						
Yes	22	91	[71–97]	85	[63–95]	0.165
No	41	84	[70–93]	75	[59–87]	
						
*External radiation therapy*						
Yes	11	91	[62–98]	79.5	[48–94]	0.98
No	52	86	[73–93]	75.6	[61–86]	
						
*Lymph node dissection*						
Complete	42	88	[74–95]	79	[63–89]	0.86
Incomplete	21	84	[62–95]	78	[55–91]	

): the stage of the disease (*P*<0.01), pT (*P*<0.001), pN (*P*<0.001), the percentage of metastatic lymph nodes (*P*<0.01) and the postoperative plasma CT level (*P*<0.001). Gender, age at diagnosis and hereditary status of the disease were not statistically significant. The results were not modified when postoperative external radiotherapy and the extent of lymph node dissection were included in the analysis.

The stage of the disease, tumour extension, node status, percentage of metastatic lymph nodes and postoperative plasma CT level were highly interrelated since all pT4 patients had lymph node metastases, all had more than 10% of metastatic lymph nodes and all except one had a detectable postoperative basal CT level. The percentage of metastatic lymph nodes and the stage of the disease were therefore excluded from the multivariate analysis which was performed with only three parameters: pT, pN and postoperative basal CT level. The independent significant prognostic parameters for imaging-detected relapse were the postoperative plasma CT level (*P*<0.01) and the tumour extension (*P*<0.05). pN was not an independent, statistically significant prognostic parameter (*P*=0.1).

**Figure 1:**
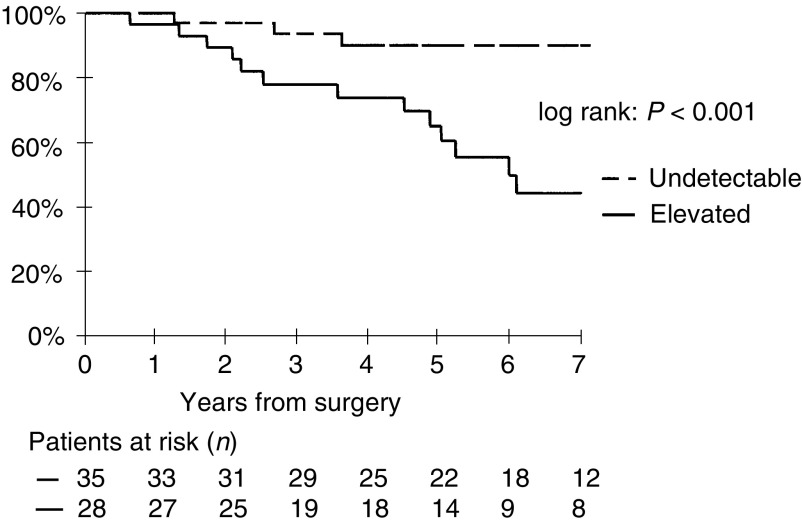
Relapse-free survival according to basal postoperative CT level.

The 3- and 5-year relapse-free survival rates were 94% (CI, 80–98%) and 90% (CI, 75–97%), respectively, in the 35 patients with an undetectable postoperative basal CT level, and 78% (CI, 59–90%) and 61% (CI, 41–77%), respectively, in the 28 patients with detectable postoperative basal CT level (*P*<0.005) (Figure 1

). An imaging-detected relapse occurred in three of the 35 patients (9%) with an undetectable postoperative basal CT level and in 15 of the 28 patients (54%) with an elevated postoperative basal CT level (Table 2). In the three patients with an undetectable postoperative CT level who relapsed, postoperative pentagastrin-stimulated CT level was undetectable in one patient and attained 43 and 162 pg ml^−1^ respectively in the other two.

**Figure 2:**
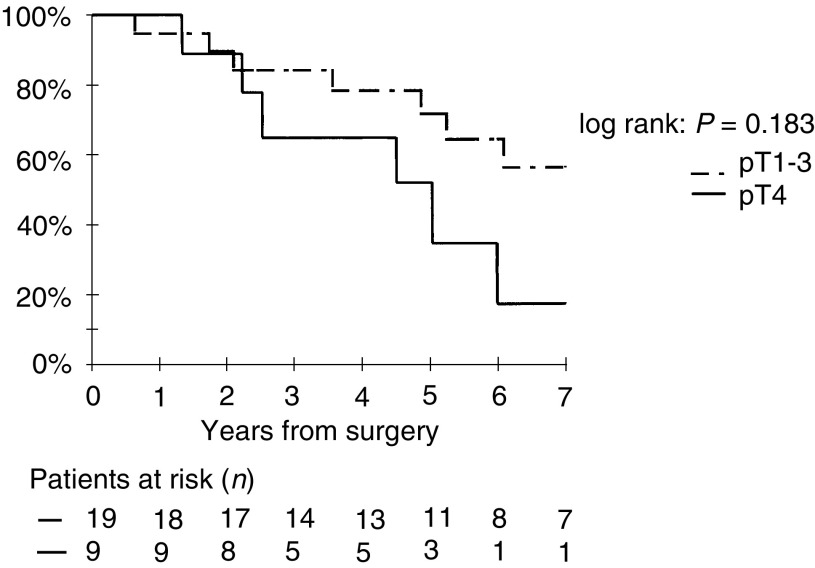
Relapse-free survival in patients with elevated basal postoperative CT level according to pT.

The 3- and 5-year relapse-free survival rates were 92% (CI, 81–87%) and 85% (CI, 72–93%), respectively, in the 53 pT1–3 patients, and 57% (CI, 28–82%) and 48% (CI, 20–74%), respectively, in the 10 pT4 patients (*P*<0.001). An imaging-detected relapse occurred in 11 patients with a pT1–3 tumour (10pT2 and 1pT3) and in seven patients with a pT4 tumour. In patients with elevated postoperative basal CT level, the 5-year relapse-free survival rate was 84% (CI, 62–95%) in pT1–3 patients and 35% (CI, 11–70%) in pT4 patients (Figure 2

). The difference is, however, not statistically significant (*P*=0.2).

The 3- and 5-year relapse-free survival rates were both 100% in the 23 pT1–3 N0 patients and 86% (CI, 69–94%) and 72% (CI, 52–86%), respectively, in the 30 pT1–3 N1 patients (*P*<0.001).

## DISCUSSION

Nonmetastatic MTC has a favourable prognosis. However, the 10-year overall survival rate varied widely among series, from 55 up to 100% respectively (Rougier *et al*, 1983; Saad *et al*, 1984; Schroder *et al*, 1988; Raue *et al*, 1993; Dottorini *et al*, 1996; Scopsi *et al*, 1996; Bergholm *et al*, 1997; Modigliani *et al*, 1998). As therapy should be adapted to the prognosis of the disease, the identification of patients at high risk of relapse is essential. In the present study on 63 patients with normal postoperative imaging and either an undetectable postoperative basal CT level or an elevated postoperative basal CT level, the 3- and 5-year overall survival rates were 98 and 97%, respectively. Imaging-detected relapse occurred in 29% of the patients, and the 3- and 5-year relapse-free survival rates were 86 and 79%, respectively. In patients with elevated postoperative basal CT level, the 5-year relapse-free survival rate was 61%, similar to that reported by van Heerden *et al* (1990).

The prognostic factors for survival of MTC patients include the age at diagnosis, the gender and the TNM classification (Schroder *et al*, 1988; Raue *et al*, 1993; Dottorini *et al*, 1996; Scopsi *et al*, 1996; Bergholm *et al*, 1997; Modigliani *et al*, 1998; Kebebew *et al*, 2000a). Data concerning MTC patients with normal postoperative imaging are few. In one study where the age was below 35 years, the presence of more than three metastatic nodes at initial surgery was found to be a significant prognostic factor for relapse at univariate analysis (van Heerden *et al*, 1990). In our study, at univariate analysis, stage of the disease, pT, pN, percentage of metastatic lymph nodes and postoperative basal CT level were found to be prognostic factors for imaging-detected relapse. Owing to a high link between stage, percentage of metastatic lymph nodes, pT and pN, multivariate analysis was performed on pT, pN and postoperative basal CT level. Only the postoperative basal CT level and pT were independent prognostic factors.

Calcitonin is a highly sensitive marker of persistent disease that is more sensitive than any other imaging modality (Frank-Raue *et al*, 1992; Abdelmoumene *et al*, 1994). Elevated postoperative basal CT level was described as a survival prognostic factor (Dottorini *et al*, 1996; Modigliani *et al*, 1998). In our study, it appeared prognostic for imaging-detected relapse. An imaging-detected relapse occurred in 54% of the patients with an elevated postoperative basal CT level and in 9% of the patients with an undetectable postoperative basal CT level. Our active follow-up permitted the early diagnosis of recurrences at a stage when basal CT level was below 150 pg ml^−1^ and CEA level was still in the normal range (five patients). In patients with postoperative undetectable basal CT level, a pentagastrin-stimulated test should be performed, when possible. However, even an undetectable pentagastrin-stimulated CT level does not totally exclude the risk of relapse, and this was observed in one of our patients, in accordance with another study (Modigliani *et al*, 1998). This underlines the need for prolonged follow-up in all MTC patients.

Tumour extension is, in our study and in others, a significant independent prognostic factor (Scopsi *et al*, 1996). In our study, in the group of patients with an elevated postoperative basal CT level, the risk of relapse was even higher when the tumour was pT4. In this group, we did not, however, show a significant difference in relapse-free survival, probably because of the very low number of patients.

Node status was identified as a prognostic factor in the univariate analysis in our study and in others (Schroder *et al*, 1988; van Heerden *et al*, 1990; Raue *et al*, 1993; Dottorini *et al*, 1996; Scopsi *et al*, 1996; Bergholm *et al*, 1997; Modigliani *et al*, 1998; Hyer *et al*, 2000; Kebebew *et al*, 2000a). In another study, with rising numbers of metastatic lymph node, gross distant metastases occurred more frequently and postoperative basal CT level was less often normalised (Machens *et al*, 2000). In our study, among pT1–3 patients, a relapse occurred in none of the pN0 patients and in 37% of the pN1 patients. Among pT4N1 patients, a relapse occurred in 70%. Taken together, these data suggested that both tumour extension and nodal involvement were prognostic indicators of relapse. However, the strong interrelation between pT and pN was responsible for a competition between these two factors in the multivariate analysis.

This study allowed us to identify patients with a high risk of relapse among those with normal postoperative imaging, in whom an aggressive therapeutic approach should be considered. In retrospective studies, external radiation therapy to the neck was associated with a decreased risk of local relapse with a controversial effect on survival (Rougier *et al*, 1983; Samaan *et al*, 1988; Fersht *et al*, 2001). In our study, external radiation therapy was not found to be a prognostic factor, but was performed only in a few patients either with pT1–3 tumour or pT4 tumour. Nevertheless, external radiation therapy should be discussed in patients with an elevated postoperative CT level and a pT4 tumour. As the response rate to cytotoxic treatments is below 20% and as responses are partial and transient (Schlumberger *et al*, 1995), there is no place for adjuvant chemotherapy in MTC patients with normal imaging. It should only be given to patients with rapidly progressive metastatic disease.
